# Navigating the Future of Cardiac Diagnostics: Insights From Artificial Neural Networks

**DOI:** 10.7759/cureus.54011

**Published:** 2024-02-11

**Authors:** Tanya Sinha, Swathi Godugu, Syed Faqeer Hussain Bokhari

**Affiliations:** 1 Medical Education, Tribhuvan University Institute of Medicine, Kathmandu, NPL; 2 General Medicine, Zaporizhzhya State Medical University, Zaporizhzhya, UKR; 3 Surgery, King Edward Medical University, Lahore, PAK

**Keywords:** machine learning, artificial intelligence, telemedicine, wearable devices, predictive analytics, cardiovascular diseases, cardiology, diagnostics, artificial neural networks

## Abstract

Cardiovascular diseases remain a leading cause of mortality globally, necessitating innovative approaches for early detection and precise diagnostic methodologies. Artificial neural networks (ANNs), inspired by the complexity of the human brain's neural networks, have emerged as powerful tools for transforming the landscape of cardiac diagnostics. ANNs are capable of learning complex patterns from data. In cardiac diagnostics, these networks are employed to analyze intricate cardiovascular data, providing insights into diseases such as coronary artery disease and arrhythmias. From personalized medicine approaches to predictive analytics, ANNs can revolutionize the identification of cardiovascular risks, enabling timely interventions and preventive measures. The integration of ANNs with wearable devices and telemedicine is poised to establish a connected healthcare ecosystem, providing holistic and continuous cardiac monitoring. However, challenges persist, including ethical considerations surrounding patient data and uncertainties in diagnostic outcomes. Looking forward, the prospects of ANNs in cardiac diagnostics are promising. Anticipated technological advancements and collaborative efforts between medical and technological communities are expected to drive innovation, address current challenges, and foster a new era of precision cardiac care.

## Editorial

Introduction

Cardiac diagnostics play a pivotal role in timely disease detection and patient outcomes. The current landscape of cardiac diagnostics involves a multitude of methods, ranging from traditional tests to advanced technologies. This introduction aims to provide an overview of the existing cardiac diagnostic landscape. In recent years, artificial intelligence (AI) and machine learning (ML) have emerged as transformative forces in healthcare. Artificial neural networks (ANNs), an emerging application of AI and ML, have particularly shown promise in cardiac diagnostics [1,2]. ANNs are computational models inspired by the human brain's neural structure, capable of learning and making predictions. The integration of ANNs in cardiac diagnostics signifies a paradigm shift in the approach toward identifying and predicting heart-related conditions.

ANNs bring a unique set of advantages to cardiac diagnostics, including enhanced accuracy and efficiency in predicting heart diseases. The utilization of various parameters, teaching methods, and network topologies has been a subject of research to optimize the accuracy of cardiac disease diagnosis. Timely diagnosis holds the potential to transform patient outcomes and reduce healthcare costs. As we explore the role of ANNs in cardiac diagnostics, it becomes evident that these technologies hold the key to more accurate and efficient identification of heart-related conditions. This editorial's primary objective is to delve into the profound impact of ANNs on advancing cardiac diagnostics. ANNs have demonstrated notable capabilities in analyzing complex patterns and extracting meaningful insights from vast datasets, which is particularly relevant in the intricate domain of cardiac health.

Fundamentals of ANNs

Artificial neural networks (ANNs) are computational models inspired by the structure and functionality of the neural networks of the human brain. ANNs consist of layers of interconnected nodes or neurons, typically organized into an input layer, hidden layers, and an output layer. Neurons in each layer are connected to neurons in adjacent layers through weighted connections. These weights are adjusted during the training phase, allowing the network to learn and adapt to patterns in data [3]. The architecture of ANNs mirrors the neural structure of the human brain, facilitating the learning and decision-making processes. While simpler than the human brain, ANNs excel in pattern recognition, making them adept at analyzing complex datasets, especially relevant in the context of cardiac diagnostics.

ANNs function through a process known as forward propagation, where input data is passed through the network and predictions are generated at the output layer. During training, a backpropagation algorithm adjusts the weights based on the prediction error, enhancing the network's accuracy over time. In the realm of cardiac diagnostics, ANNs process various patient data such as medical history, test results, and lifestyle factors. Studies have explored their effectiveness in diagnosing coronary artery disease, myocardial infarction, and other cardiac disorders [1,2,4,5]. This comprehensive approach enables them to identify subtle patterns indicative of cardiac pathology. Their ability to adapt to evolving datasets enhances diagnostic precision. Compared to traditional diagnostic methods in cardiology, ANNs offer a more dynamic and adaptable approach. Traditional methods may rely on predetermined algorithms and predefined criteria while ANNs learn from data patterns, potentially uncovering nuanced insights that conventional methods might overlook. This adaptability positions ANNs as valuable tools in the evolving landscape of cardiac diagnostics.

Integration of ANNs in cardiac diagnostics

The seamless integration of ANNs into cardiac diagnostics represents a transformative phase in medical technology. This section explores the crucial steps and notable aspects associated with the incorporation of ANNs in cardiac diagnostic processes. The foundation of effective cardiac diagnostics using ANNs lies in the comprehensive collection of relevant cardiac data. Diverse datasets encompassing patient medical history, test results, and lifestyle factors are crucial for training ANNs to recognize intricate patterns indicative of various heart conditions. Before feeding data into ANNs, rigorous preprocessing steps are undertaken to ensure data quality. Normalization and feature scaling are common techniques applied to maintain consistency and facilitate the network's ability to extract meaningful insights. This step is crucial for enhancing the overall performance and reliability of the diagnostic process.

In the realm of cardiac diagnostics, supervised learning is employed to enhance diagnostic accuracy. ANNs are trained on labeled datasets, associating specific input patterns with corresponding output labels. This process enables the network to learn and make accurate predictions when presented with new, unseen data. The supervised learning approach has demonstrated success in various studies focused on heart disease prediction. Beyond supervised learning, unsupervised learning plays a pivotal role in pattern recognition within cardiac diagnostics. Unsupervised algorithms allow ANNs to identify hidden patterns and relationships in data without predefined labels. This capability is particularly valuable when dealing with complex and unstructured datasets, contributing to a more holistic understanding of cardiac conditions.

Several notable case studies exemplify the success of ANNs in cardiac diagnostics. For instance, the utilization of deep neural networks combined with case-based reasoning has shown promising results in the classification of coronary artery disease, achieving high accuracy rates [1,4]. Another study highlights the use of distinct artificial intelligence techniques, including ANNs, for heart disease prediction, showcasing the potential for accurate risk assessment [5]. In comparison to traditional diagnostic methods in cardiology, ANNs exhibit superior adaptability and dynamic learning capabilities. While traditional methods often rely on predetermined algorithms, ANNs can uncover nuanced insights by adapting to evolving datasets. Comparative analyses underscore the potential of ANNs to outperform traditional methods in terms of accuracy and efficiency, providing a compelling argument for their integration into mainstream cardiac diagnostics.

Advantages and challenges

The incorporation of ANNs in cardiac diagnostics heralds both substantial advantages and notable challenges, shaping the landscape of modern medical technology. One of the primary advantages of employing ANNs in cardiac diagnostics is the remarkable enhancement in diagnostic accuracy and efficiency. ANNs, through their ability to process vast datasets and recognize intricate patterns, contribute to the more precise identification of cardiac conditions. This heightened accuracy translates into improved patient outcomes and facilitates tailored treatment strategies. Studies have demonstrated the superiority of ANN-based diagnostic models, showcasing their potential to outperform traditional methods. The utilization of ANNs opens avenues for early detection and intervention in cardiac ailments. By leveraging ML algorithms, ANNs can analyze diverse patient data to identify subtle indicators of potential cardiovascular issues. This early detection capability empowers healthcare professionals to initiate interventions at a stage where interventions can be more effective, potentially preventing the progression of cardiac diseases and reducing the overall burden on healthcare systems.

Despite their potential benefits, the integration of ANNs in cardiac diagnostics raises ethical considerations, particularly regarding the utilization of patient data. The ethical dimensions encompass issues of consent, privacy, and the responsible handling of sensitive medical information. Patient privacy stands at the forefront of ethical considerations, as the utilization of large datasets, often containing intimate health details, becomes integral to the functioning of neural networks. Striking a balance between leveraging these datasets for diagnostic precision and ensuring the confidentiality of individual health records is paramount. Researchers and healthcare providers must adopt stringent measures to anonymize and protect patient data, thus mitigating the risks associated with unauthorized access or data breaches. To address patient privacy concerns, the establishment of robust regulatory frameworks and compliance measures is imperative. Legal standards and guidelines must be in place to govern the collection, storage, and utilization of health data in the realm of cardiac diagnostics employing ANNs. Adherence to established regulations not only ensures the protection of patient privacy but also engenders trust among individuals contributing to medical datasets. This trust is fundamental for the sustained development and ethical application of ANN-driven diagnostic technologies. Informed consent and transparent communication with patients regarding the utilization of their data are essential. Healthcare providers must elucidate the nature of AI-driven diagnostics, the potential implications, and the measures in place to protect patient privacy. This transparency fosters a collaborative and informed healthcare environment, allowing individuals to make decisions regarding their participation in AI-enhanced diagnostic initiatives based on a comprehensive understanding of the processes involved. While ANNs showcase impressive capabilities, the interpretability of their decision-making processes also poses challenges. Understanding how ANNs arrive at specific diagnostic conclusions is complex, creating a potential barrier to widespread acceptance and trust in these technologies. Furthermore, uncertainties regarding the generalization of ANN models across diverse patient populations and healthcare settings present ongoing challenges for their integration into mainstream clinical practice.

Future prospects

As we embark on the future of cardiac diagnostics, the integration of ANNs presents exciting possibilities and holds the promise of revolutionizing the landscape. The trajectory of ANN technology in the context of cardiac diagnostics is poised for significant improvements. With ongoing research and development, we anticipate enhanced model architectures, optimization algorithms, and training techniques. Advancements in hardware such as more powerful graphics processing units (GPUs) and tensor processing units (TPUs) will further accelerate the computation capabilities of ANNs, allowing for quicker and more efficient processing of complex cardiac data. These improvements are expected to translate into higher accuracy and faster diagnostic outcomes, ultimately benefiting patient care. These technologies are projected to play a pivotal role in personalized medicine, tailoring diagnostic approaches to individual patient profiles. Predictive analytics powered by ANNs could enable the identification of cardiovascular risks at an early stage, facilitating timely interventions and preventive measures. Furthermore, the integration of ANNs with other emerging technologies, such as wearable devices and telemedicine, is expected to create a holistic and connected healthcare ecosystem.

The intersection of ANNs and cardiology is a dynamic field with various ongoing and prospective research directions. Current efforts focus on refining diagnostic models for specific cardiac conditions, optimizing interpretability, and addressing data privacy concerns. Future research is likely to explore the integration of multi-modal data, including genetic and lifestyle factors, to enhance the predictive power of ANNs. The realization of the full potential of ANNs in cardiac diagnostics necessitates collaborative efforts between the medical and technological communities. Clinicians, data scientists, and engineers must work hand-in-hand to ensure the development of clinically relevant and ethically sound diagnostic tools. This collaboration involves aligning technological advancements with the practical needs of healthcare providers, promoting interdisciplinary research, and fostering a shared vision for the future of cardiac care.
